# The endoplasmic reticulum stress status of CD4+ T lymphocytes and its association with mTOR-mediated autophagic-lysosomal disorder in elderly sepsis patients

**DOI:** 10.3389/fimmu.2025.1648075

**Published:** 2025-08-26

**Authors:** Wei Cheng, Jiahui Zhang, Dongkai Li, Yawen Xie, Xianli Lei, Hao Wang, Na Cui

**Affiliations:** ^1^ Department of Critical Care Medicine, State Key Laboratory of Complex Severe and Rare Diseases, Peking Union Medical College Hospital, Chinese Academy of Medical Science and Peking Union Medical College, Beijing, China; ^2^ Department of Critical Care Medicine, Beijing Jishuitan Hospital, Capital Medical University, Beijing, China; ^3^ Department of Critical Care Medicine, Beijing Chao-Yang Hospital, Capital Medical University, Beijing, China

**Keywords:** endoplasmic reticulum stress, sepsis, elderly, mTOR, autophagiclysosomal disorder

## Abstract

**Aims:**

to clarify the endoplasmic reticulum stress (ERS) status of CD4+ T lymphocytes in sepsis patients, particularly elderly individuals aged over 65 years, and to elucidate its association with mTOR-mediated autophagic-lysosomal disorder.

**Methods:**

62 sepsis patients were enrolled from January 1 to July 31, 2024. Peripheral blood mononuclear cells (PBMCs) were isolated within 24 hours post-enrollment. flow cytometry was used to quantify the expression levels of ERS markers (CHOP and GRP78) and mTOR-mediated autophagic-lysosomal fusion markers (mTOR, LC3II and P62) on CD4+ T lymphocytes. These markers were compared between sepsis and non-sepsis patients, elderly and non-elderly sepsis patients, survivors and non-survivors based on in-hospital mortality. The correlations between CHOP mean fluorescence intensity (MFI) and CD4+ T lymphocyte count, LC3II MFI, and P62 MFI were also analyzed.

**Results:**

Compared to non-septic controls, sepsis patients exhibited significantly higher CHOP and GRP78 MFIs (210.9 versus 142.9, P<0.001 and 279.1 versus 223.7, P=0.045). Within the sepsis group, elderly patients and non-survivors showed significantly higher CHOP and GRP78 MFIs (334.4 versus 164.2, P<0.001 and 374.3 versus 218.6, P<0.001; 390.8 versus 177.6, P<0.001 and 389.1 versus 227.0, P<0.001). CHOP MFI on CD4+ T lymphocytes showed significant correlations with LC3II and P62 MFIs in sepsis patients (Pearson’s correlation r=0.657, p<0.001 and r=0.811, P<0.001), elderly sepsis patients (r=0.644, P<0.001 and r=0.710, P<0.001), and non-survived elderly sepsis patients (r=0.897, P<0.001 and r=0.772, P=0.009).

**Conclusion:**

ERS in CD4+ T cells was enhanced in sepsis patients, particularly in elderly and non-survived individuals; ERS is strongly associated with mTOR-mediated autophagic-lysosomal disorder.

**Clinical trial registration:**

chictr.org.cn, identifier ChiCTR2300074175.

## Background

Till now, sepsis remains a leading cause of morbidity and mortality globally, accounting for 20% of deaths worldwide (1, 2). While sepsis affects all the age groups, its incidence increases significantly with advancing age. A nationwide survey in China demonstrated that hospitalized sepsis incidence was 328, 359 and 422 cases per 100,000 from 2017 to 2019, with 57.5% of cases occurring in elderly patients over 65 years (3). Age is an independent risk factor for sepsis-related mortality and morbidity (4–6), and age-specific therapeutic approaches are urgent to improve clinical outcomes. Sepsis is defined as life-threatening organ dysfunction caused by an imbalanced host response to infection, where the interplay of hyperinflammation and immunosuppression drives pathogenesis and influences prognosis (7, 8). Immune dysfunction is particularly pronounced in elderly patients and contributes significantly to poor prognosis (5, 9). Understanding the molecular regulation mechanism of immune cells in elderly sepsis patients could enhance early warning and prognosis prediction, potentially providing targets for immune intervention.

T cells are the primary effectors of anti-infection response during sepsis, and their impaired proliferation and differentiation are critical factors for the poor prognosis of elderly sepsis patients. Apoptosis is a key contributor to T cell depletion in sepsis, with multiple factors implicated in this process (10). The endoplasmic reticulum (ER) is a vital organelle responsible for protein translation, processing, and modification. Accumulation of waste products disrupts ER homeostasis, leading to ER stress (ERS) (11). ERS is a key regulator of T cell metabolism in sepsis and promotes sepsis-associated immunosuppression (SAI) by inducing CD4+ T cell apoptosis (10, 12). Age-related declines in ER function exacerbate ERS during sepsis, leading to T cell apoptosis, organ damage and subsequent poor clinical prognosis (9, 12, 13). However, there has been little research on the correlation between ERS and T cell immunity in elderly sepsis.

The mammalian target of rapamycin (mTOR) is a serine/threonine protein kinase that regulates cell proliferation, differentiation, and autophagy (14). Targeting the mTOR pathway for T cell modulation has shown promise in autoimmune diseases and cancer therapy, with studies demonstrating that mTOR inhibition can mitigate SAI, reduce organ damage, and improve sepsis outcomes (15–17). Our previous research identified mTOR as a key regulator of T cell metabolism (18, 19) and demonstrated its role in modulating ERS-induced CD4+ T cell apoptosis during sepsis (20). Our recent preclinical study showed that the mTOR pathway mediates ERS-induced apoptosis of CD4+T cells by inhibiting autophagosome–lysosome fusion in a septic mouse model (Lei et al, in press (21)). So, we hypothesized that mTOR pathway could regulate the intensity of ERS apoptosis through modulating autophagy in elderly sepsis patients, and played important roles in the occurrence of SAI and severely affected the prognosis of these patients.

This clinical study was designed to evaluate the intensity of ERS and its associated CD4+ T cell apoptosis in elderly sepsis patients, and explore the correlation between mTOR-mediated autophagic-lysosomal disorder and ERS, providing evidence for future preclinical and clinical research.

## Materials and methods

### Study population

This prospective study was conducted at Peking Union Medical College Hospital from January 1 to July 31, 2024 and registered at the Chinese Clinical Trial Registry (chictr.org.cn; identifier: ChiCTR2300074175). Patients admitted to intensive care unit (ICU) were enrolled if they met the following criteria: (1) age≥18 years; (2) ICU stay≥24 hours; and (3) met the 2016 sepsis criteria (8, 22). Sepsis was defined as life-threatening organ dysfunction (identified by an acute increase in total Sequential Organ Failure Assessment (SOFA) score ≥2 points) caused by a dysregulated host response to infection. Septic shock was defined as persisting hypotension requiring vasopressors to maintain a mean arterial pressure ≥65 mmHg and a serum lactate level >2 mmol/L despite adequate fluid resuscitation (8, 22). Infection was defined on the basis of clinical features, laboratory findings, and imaging tests based on the criteria of the international sepsis forum consensus conference (23). Patients were excluded if they met any one of the following criteria: (1) age<18 years; (2) pregnant or breastfeeding; (3) receiving chemotherapy, radiation, or targeted therapy for unhealed tumors; (4) receiving glucocorticoids or immunosuppressants for autoimmune diseases; (5) acquired immunodeficiency, or hematological disorders involving white blood cells; or (6) end-stage patients admitted for palliative care only. All the enrolled sepsis patients were divided into elderly (aged≥65 years) and non-elderly (aged<65 years) groups (3, 6, 9). Non-septic patients admitted to our ICU during the same period were enrolled as controls. The study was approved by the PUMCH institutional review board (approval number: I-24PJ1761), and all procedures were performed in compliance with relevant laws and institutional guidelines. The work has been carried out in accordance with The Code of Ethics of the World Medical Association (Declaration of Helsinki) for experiments involving humans and informed consent was obtained from all the patients or their next of kin at ICU admission.

### Study method

Baseline characteristics, vital signs, laboratory tests, and treatments on the first day of ICU admission were recorded. Acute Physiology and Chronic Health Evaluation (APACHE) II and SOFA scores were calculated. Patients were followed up for 28 days post-enrollment and classified as survivors or non-survivors based on 28-day mortality.

**Figure 1:**
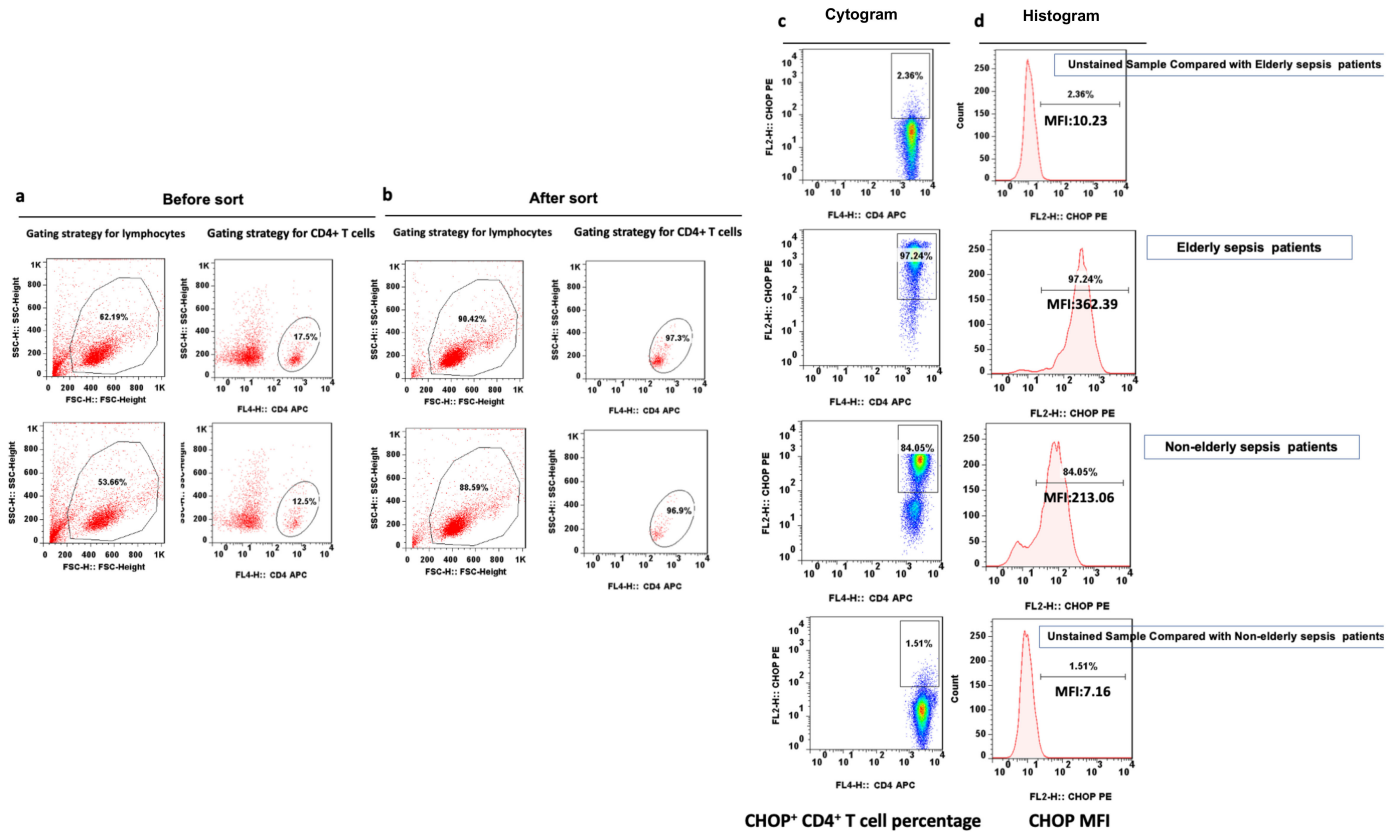
Representative flow dot plots of lymphocyte gating strategy. The lymphocyte **(a)**, CD4+ T lymphocyte **(b)**, percentage of CHOP+ CD4+ T lymphocyte **(c)** and CHOP MFI on CD4+ T lymphocyte **(d)** between elderly and non-elderly sepsis patients. MFI mean fluorescent intensity, CHOP PERK-mediated C/EBP homologous protein.

Flow cytometric analysis was performed on peripheral blood mononuclear cells (PBMCs) isolated from EDTA-anticoagulated blood using Ficoll density-gradient centrifugation. CD4+ T cells were positively selected using CD4 MicroBeads (Miltenyi Biotec), with post-sort purity (>95%) verified by flow cytometry (anti-CD4-FITC, BioLegend). Both pre-sort PBMCs and post-sort CD4+ T cell populations are shown in **Figure 1**. Cells were stained with fluorochrome-conjugated antibodies against surface markers (CD3, CD4, CD8) and intracellular markers (IFN-γ for Th1, IL-4 for Th2, IL-17 for Th17, and Foxp3/CD25 for Tregs), followed by fixation/permeabilization (Foxp3 Buffer Set, Invitrogen). Gating strategies excluded debris (FSC-H/SSC-H) and selected lymphocytes before subset analysis. Compensation used single-stain controls, with thresholds validated via fluorescence-minus-one (FMO) controls. Data were acquired on a Cytek Aurora flow cytometer (10,000 events/sample) and analyzed using FlowJo v10.1r5, reporting percentages and median fluorescence intensity (MFI) (isotype-corrected) (24, 25). We additionally quantified the percentages and MFIs of ERS markers (CHOP, GRP78) and autophagic-lysosomal fusion markers (mTOR, LC3I, LC3II, and P62) on CD4+ lymphocytes (**Figure 1**).

### Statistics

Normality of data distribution was assessed using Kolmogorov-Smirnov test, with graphical evaluation by Q-Q plots. Data were presented as mean ± standard deviation (SD) (normally distributed) or median (interquartile range, IQR). Group comparisons used Student’s t-test or Mann-Whitney U test. Categorical variables were analyzed via chi-squared test or Fisher’s exact test. Pearson’s correlation assessed association between ERS and autophagic-lysosomal disorder biomarkers. Statistical significance was set at P<0.05 (SPSS V25.0 Chicago, Illinois). Due to the exploratory nature of this study, sample size was empirically determined based on previous similar experiments.

## Results

### Baseline characteristics of enrolled sepsis patients and comparison with non-septic critically ill patients

During the study period, a total of 62 sepsis patients were enrolled (**Supplementary File S1**), with a median age of 63 years (IQR: 51–71), and 56.5% were male (35 cases). The sepsis cohort exhibited significantly higher APACHE II (19 versus 8, P<0.0001) and SOFA scores (8 versus 5, P=0.011), elevated lactate levels (3.6 versus 2.0 mmol/L, P<0.0001), lower albumin level (32 versus 36g/L, P<0.001), higher APTT-R (1.2 versus 1.0, P<0.001) on admission, and increased 28-day mortality (21.0% versus 0%, P=0.039) but shorter hospital stay (10 versus 19 days, P=0.001). The percentage of CD4+ T cells expressing CHOP, LC3II was significantly higher in septic patients than in non-septic critically ill patients (92.4% versus 64.1%, P<0.001 and 47.2% versus 23.7%, P=0.004) and the MFI of LC3II, P62, CHOP, and GRP78 on CD4+ cells were also significantly higher (152.7 versus 99.6, P<0.001; 246.6 versus 177.2, P<0.001; 92.4 versus 64.1, P<0.001; and 279.1 versus 223.7, P=0.045, respectively) (**Supplementary File S2**).

### Comparison between elderly and non-elderly sepsis patients

Among the 62 sepsis patients, 29 were classified as elderly (aged≥65 years) with a median age of 71 years (IQR 69-75), and 33 were non-elderly (aged<65 years) with a median age of 52 years (IQR:46-59). Elderly sepsis patients had higher rates of chronic kidney disease (20.7% versus 3.0%, P=0.028), longer ICU and hospital stays (7 versus 3 days, P=0.006; 14 versus 8 days, P<0.001 respectively), higher APACHE II and SOFA scores (24 versus 19, P =0.042; 10 versus 6, P=0.005) and higher in-hospital mortality rate (34.5% versus 9.1%, P=0.014) (**Table 1**). There were no significant differences in the clinical parameters at ICU admission, the initial treatment and antibiotics on the first day (**Supplementary File S3**).

**Table 1:** Comparison of baseline characteristics between elderly and non-elderly sepsis patients.

	All sepsis (N=62)	Elderly sepsis* (N=29)	Non-elderly sepsis (N=33)	P
Age (years)	63 (51, 71)	71 (69, 75)	52 (46, 59)	<0.001
Sex (Male n, %)	35 (56.5%)	15 (51.7%)	20 (60.6%)	0.482
Transferred from				0.568
Ward	9 (14.5%)	5 (17.2%)	4 (12.1%)	
Emergency Room	53 (85.5%)	24 (82.8%)	29 (87.9%)	
Comorbidities				
Chronic heart disease	22 (35.5%)	10 (34.5%)	12 (36.4%)	0.877
COPD	5 (8.1%)	3 (10.3%)	2 (6.1%)	0.536
Diabetes Mellitus	20 (32.3%)	12 (41.4%)	8 (24.2%)	0.150
Chronic kidney disease	7 (11.3%)	6 (20.7%)	1 (3.0%)	0.028
Chronic liver disease	4 (6.5%)	2 (6.9%)	2 (6.1%)	0.894
Solid Tumor	17 (27.4%)	7 (24.1%)	10 (30.3%)	0.587
Autoimmune Disease	10 (16.1%)	7 (24.1%)	3 (9.1%)	0.108
Hematologic Disease	3 (4.8%)	3 (10.3%)	2 (6.1%)	0.536
Infection sites				0.349
Pulmonary	34 (54.8%)	16 (55.2%)	18 (54.5%)	
Bloodstream infection	2 (3.2%)	2 (6.9%)	0	
Abdominal	23 (37.1%)	9 (31.0%)	14 (42.4%)	
SSTI	3 (4.8%)	2 (6.9%)	1 (3.0%)	
Apache II score	19 (16, 28)	24 (17, 30)	19 (15, 25)	0.042
SOFA score	8 (5, 11)	10 (7, 12)	6 (4, 9)	0.005
Hospital Stay (days)	10 (8, 16)	14 (10, 24)	8 (5, 14)	<0.001
ICU Stay (days)	5 (3, 11)	7 (4, 13)	3 (2, 8)	0.006
In-hospital Mortality (n, %)	13 (21%)	10 (34.5%)	3 (9.1%)	0.014

*All the enrolled sepsis patients were divided into elderly (aged≥65 years) and non-elderly (aged<65 years) groups.

Values are presented as median and interquartile range (IQR) for continuous variables or as number of cases and percentage for categorical data. COPD, chronic obstructive pulmonary disease; SSTI, skin and soft tissue infection; Apache II score, acute physiology and chronic health evaluation II score; SOFA, score, subsequent organ failure assessment score; ICU, intensive care unit.

The serum Interleukin 6 (IL-6) level was significant higher in elderly sepsis patients (128 versus 43.8pg/ml, P=0.043). Compared with non-elderly sepsis patients, the monocyte count, Lymphocyte count, B lymphocyte count and T lymphocyte count were all significant lower in elderly sepsis patients (380 versus 600, P=0.017; 448 versus 1147, P<0.001; 79 versus 190, P<0.001; 326 versus 799, P<0.001, respectively). The CD4+ T cell count, CD4+CD28+ T cell count, CD8+ T cell count and CD8+CD28+ T cell count were also significantly lower in elderly sepsis patients (194 versus 474, P<0.001; 180 versus 451, P=0.001;107 versus 199, P<0.001; 35 versus 132, P<0.001 respectively). The percentage of circulating CD4+ T lymphocytes expressing LC3II and CHOP were significantly higher in elderly sepsis patients (71.1% versus 38.7%, P=0.003; 95.5% versus 81.1%, P=0.004). When flow cytometry data were expressed as MFI, the MFIs of mTOR, LC3I, LC3II, P62, CHOP, and GRP78 on CD4+ T lymphocytes were all significantly higher in elderly sepsis patients than in non-elderly sepsis patients (199.1 versus 139.2, P=0.026; 173.4 versus 103.8, P=0.023; 236.7 versus 123.5, P<0.001; 318.4 versus 192.5, P<0.001; 334.4 versus 164.2, P<0.001; 374.3 versus 218.6, P<0.001 respectively) (**Table 2**, **Supplementary File S4**).

**Table 2:** Comparison of inflammatory factors, T lymphocyte subsets and markers on CD4^+^ T cells between elderly and non-elderly sepsis patients.

	All sepsis (N=62)	Elderly sepsis* (N=29)	Non-elderly sepsis (N=33)	P
Inflammatory factors (median and IQR)				
C3 (g/L)	0.8 (0.7, 1.0)	0.8 (0.7, 1.0)	0.9 (0.7, 1.1)	0.467
C4 (g/L)	0.2 (0.1, 0.2)	0.2 (0.2, 0.2)	0.2 (0.1, 0.2)	0.302
IgG (g/L)	7.9 (5.6, 10.6)	7.9 (6.3, 11.6)	8.1 (4.8, 9.9)	0.386
IgA (g/L)	2.1 (1.3, 2.9)	2.3 (1.5, 2.7)	1.9 (1.2, 2.9)	0.521
IgM (g/L)	0.6 (0.4, 0.9)	0.6 (0.4, 0.7)	0.6 (0.4, 0.9)	0.363
IL-6 (pg/ml)	75.8 (34.5, 334.3)	128.0 (45.9, 674.0)	43.8 (32.8, 112.0)	0.043
IL-8 (pg/ml)	49.0 (30.8,128.5)	62.0 (34.0, 161.0)	45.0 (28.5, 97.5)	0.138
IL-10 (pg/ml)	7.9 (5.0, 19.0)	10.6 (5.0, 31.7)	6.2 (5.0, 15.4)	0.225
TNF-a (pg/ml)	19.1 (12.3, 29.2)	19.4 (13.8, 34.1)	18.0 (11.5, 25.4)	0.131
T lymphocyte subsets (median and IQR) (/uL)				
White Blood Cell Count	11835 (7785, 16820)	7820 (7085, 15935)	14050 (9635, 18255)	0.085
Monocyte Count	450 (250, 710)	380 (205, 595)	600 (405, 840)	0.017
Lymphocyte Count	625 (392, 1171)	448 (365, 647)	1147 (521, 1384)	<0.001
B Lymphocyte	116 (63, 219)	79 (41, 123)	190 (89, 305)	<0.001
NK T cell Count	57 (32, 93)	51 (17, 76)	67 (42, 107)	0.045
T Lymphocyte Count	425 (261, 873)	326 (224, 444)	799 (411, 989)	<0.001
CD4+ T cell Count	260 (152, 567)	194 (135, 279)	474 (197, 666)	<0.001
CD8+ T cell Count	145 (89, 248)	107 (77, 135)	199 (146, 365)	<0.001
CD4+CD28+ T cell	242 (137, 553)	180 (105, 279)	451 (187, 611)	0.001
CD8+CD28+ T cell	72 (32, 159)	35 (19, 68)	132 (67, 204)	<0.001
CD4+/CD8+ cell ratio	1.8 (1.2, 2.9)	1.8 (1.4, 3.3)	1.8 (1.2, 2.8)	0.434
Markers on CD4^+^ T cells (median and IQR)				
mTOR (%)	81.4 (60.2, 90.9)	81.3 (62.9, 90.3)	81.5 (59.7, 92.1)	1.000
mTOR MFI	166.2 (114.4, 306.4)	199.1 (140.9, 334.8)	139.2 (86.9, 257.9)	0.026
LC3I (%)	29.0 (12.9, 56.7)	22.2 (9.7, 40.7)	36.4 (13.7, 73.5)	0.268
LC3I MFI	148.8 (84.6, 256.3)	173.4 (124.9, 263.4)	103.8 (65.5, 252.3)	0.023
LC3II (%)	47.2 (25.6, 85.7)	71.1 (34.2, 91.0)	38.7 (19.4, 61.3)	0.003
LC3II MFI	152.7 (100.9, 236.9)	236.7 (128.8, 294.4)	123.5 (90.1, 165.9)	<0.001
P62 (%)	95.7 (90.1, 97.6)	96.2 (93.7, 97.6)	94.8 (84.8, 97.6)	0.133
P62 MFI	246.6 (183.7, 340.8)	318.4 (252.5, 369.9)	192.5 (166.3, 270.4)	<0.001
CHOP (%)	92.4 (74.3, 97.6)	95.5 (88.2, 98.2)	81.1 (65.7, 96.9)	0.004
CHOP MFI	210.9 (159.7, 361.7)	334.4 (274.3, 401.4)	164.2 (134.5, 202.4)	<0.001
GRP78 (%)	88.0 (77.7, 96.1)	90.2 (82.9, 96.9)	83.0 (73.7, 95.6)	0.116
GRP78 MFI	279.1 (198.7, 383.4)	374.3 (305.9, 418.4)	218.6 (180.5, 279.1)	<0.001

*All the enrolled sepsis patients were divided into elderly (aged≥65 years) and non-elderly (aged<65 years) groups. Values are presented as median and interquartile range (IQR) for continuous variables or as number of cases and percentage for categorical data.

IQR, interquartile range; Ig, immunoglobulin; IL, interleukin; TNF-α, tumor necrosis factor α; NK T cells, natural killer T cells; IFN-r, interferon r; MFI, mean fluorescent intensity; mTOR, mammalian target of rapamycin; LC3I microtubule-associated protein light chain 3 type I; LC3II microtubule-associated protein light chain 3 type II; CHOP PERK-mediated C/EBP homologous protein.

### Comparison between survivors with non-survivors of all the sepsis patients and comparison of elderly sepsis patients based on in-hospital mortality

All the sepsis patients were divided into survivors and non-survivors based on in-hospital mortality. The non-survivors were older (70 versus 60 years old, P=0.027), had higher rates of chronic heart disease (CHD) (69.2% versus 26.5%, P=0.004) and chronic kidney disease (30.8% versus 6.1%, P=0.013), higher Apache II and SOFA scores (30 versus 19, P<0.001 and 11 versus 7, P<0.001 respectively), and longer hospital stay (15 versus 9 days, P=0.016). In the non-survivor group, serum lactate, IL-6 and IL-8 levels at ICU admission were also higher (5.5 versus 3.2 mmol/L, P<0.0001; 299.0 versus 52.6pg/ml, P=0.015 and 148.0 versus 45.0, P=0.005). The lymphocyte count, T lymphocyte count, CD4+ T cell count, CD4+CD28+ T cell count and CD8+CD28+ T cell count were all significantly lower in the non-survived sepsis group (454 versus 731/uL, P=0.013; 283 versus 597/uL, P=0.009; 152 versus 394/uL, P=0.005; 146 versus 353/uL, P=0.006; and 29 versus 81/uL, P=0.007 respectively). The percentages of CD4+ T lymphocytes expressing LC3II, CHOP and GRP78 were markedly higher than in the survivors (85.2% versus 41.6%, P=0.013; 98.3 versus 86.5%, P<0.001 and 96.4% versus 85.3%, P=0.035 respectively). The MFIs of LC3II, P62, CHOP and GRP78 on CD4+ lymphocytes were also higher in the non-surviving sepsis patients (249.9 versus 134.8, P<0.001; 323.7 versus 200.6, P=0.002; 390.8 versus 177.6, P<0.001 and 389.1 versus 227.0, P<0.001) (**Table 3**, **Supplementary File S5**).

**Table 3:** Differences between survivors and non-survivors of all the sepsis patients based on in-hospital mortality.

	All sepsis (N=62)	Non-survivors (N=13)	Survivors* (N=49)	P
Differences of baseline characteristics				
Age	63 (51, 71)	70 (65, 74)	60 (51, 70)	0.027
Age (>=65years)	29 (46.8%)	10 (76.9%)	19 (38.8%)	0.014
Comorbidities				
CHD	22 (35.5%)	9 (69.2%)	13 (26.5%)	0.004
CKD	7 (11.3%)	4 (30.8%)	3 (6.1%)	0.013
Apache II score	19 (16, 28)	30 (26, 34)	19 (16, 24)	<0.001
SOFA score	8 (5, 11)	11 (9, 13)	7 (4, 11)	<0.001
Hospital Stay	10 (8, 16)	15 (11, 26)	9 (7, 15)	0.016
Differences of clinical parameters at ICU admission				
Temperature (°C)	36.9 (36.3, 37.2)	36.2 (36.0, 37.1)	37.0 (36.5, 37.3)	0.022
Lactate (mmol/L)	3.6 (2.8, 4.9)	5.5 (4.9, 6.9)	3.2 (2.7, 3.9)	0.028
Creatinine (umol/L)	84.5 (52.5, 148.5)	148.0 (107.5, 237.5)	74.0 (47.5, 111.5)	0.007
Prothrombin Time (s)	13.8 (12.5, 15.3)	16.3 (12.6, 17.4)	13.6 (12.5, 14.7)	0.046
Differences of inflammatory factors				
C3 (g/L)	0.8 (0.7, 1.0)	0.7 (0.6, 1.0)	0.9 (0.7, 1.0)	0.027
IL-6 (pg/ml)	75.8 (34.5, 334.3)	299.0 (68.8, 1000.0)	52.6 (31.3, 133.0)	0.015
IL-8 (pg/ml)	49.0 (30.8,128.5)	148.0 (55.5, 380.5)	45.0 (27.0, 97.5)	0.005
Differences of T lymphocyte subsets (median and IQR) (/uL)				
Lymphocyte Count	625 (392, 1171)	454 (345, 593)	731 (397, 1312)	0.013
NK T cell Count	57 (32, 93)	23 (11, 53)	60 (45, 100)	0.008
T Lymphocyte Count	425 (261, 873)	283 (199, 416)	597 (286, 925)	0.009
CD4+ T cell Count	260 (152, 567)	152 (114, 240)	394 (161, 626)	0.005
CD4+CD28+ T cell	242 (137, 553)	146 (84, 225)	353 (152, 582)	0.006
CD8+CD28+ T cell	72 (32, 159)	29 (14, 90)	81 (37, 170)	0.007
Differences of markers on CD4^+^ T cells (median and IQR)				
mTOR (%)	81.4 (60.2, 90.9)	70.3 (39.4, 82.4)	85.6 (60.6, 92.6)	0.048
LC3II (%)	47.2 (25.6, 85.7)	85.2 (75.2, 297.2)	41.6 (22.9, 75.4)	0.013
LC3II MFI	152.7 (100.9, 236.9)	249.9 (181.5, 280.4)	134.8 (96.9, 184.7)	<0.001
P62 MFI	246.6 (183.7, 340.8)	323.7 (306.5, 357.5)	200.6 (174.2, 309.1)	0.002
CHOP (%)	92.4 (74.3, 97.6)	98.3 (95.6, 98.9)	86.5 (71.5, 96.2)	<0.001
CHOP MFI	210.9 (159.7, 361.7)	390.8 (313.6, 420.9)	177.6 (148.9, 321.1)	<0.001
GRP78 (%)	88.0 (77.7, 96.1)	96.4 (84.1, 98.0)	85.3 (76.5, 95.1)	0.035
GRP78 MFI	279.1 (198.7, 383.4)	389.1 (311.2, 433.1)	227.0 (191.6, 370.9)	<0.001

*All the enrolled sepsis patients were divided into survivor and non-survivor groups according to in-hospital mortality. Values are presented as median and interquartile range (IQR) for continuous variables or as number of cases and percentage for categorical data. CHD, chronic heart disease; CKD, chronic kidney disease; Apache II score acute physiology and chronic health evaluation score II; SOFA, subsequent organ failure assessment score; ICU, intensive care unit; IL, interleukin; NK T cells, natural killer T cells; MFI, mean fluorescent intensity; mTOR, mammalian target of rapamycin; LC3I microtubule-associated protein light chain 3 type I; LC3II microtubule-associated protein light chain 3 type II; CHOP PERK-mediated C/EBP homologous protein.

Subsequently, patients in the elderly sepsis group were also divided into survivors and non-survivors. The non-survivors had higher rates of CHD (60% versus 21.1%, P=0.036), higher Apache II score (30 versus 19, P=0.016), higher serum lactate (5.4 versus 2.8mmol/l, P<0.001) and creatinine (180.5 versus 71.0umol/L) levels at ICU admission. In the non-survived elderly sepsis patients, the percentages of CD4+ T lymphocytes expressing CHOP was markedly higher (98.2% versus 93.2%, P=0.021), and the percentage expressing LC3II, P62 and GRP78 was also slightly higher (87.0% versus 66.5%, P=0.128; 96.5% versus 95.7%, P=0.450 and 96.9% versus 86.8%, P=0.128 respectively). The MFIs of CHOP and GRP78 on CD4+ lymphocytes were statistically higher in the non-surviving elderly sepsis patients (398.7 versus 318, P=0.021 and 418.5 versus 329.0, P=0.021 respectively), while the MFIs of LC3II and P62 were also slightly higher (257.5 versus185.4, P=0.128 and 322.9 versus 294.8, P=0.450 respectively) (**Supplementary File S6**).

### Correlation between CHOP and markers involved in mTOR pathway mediated autophagic–lysosomal disorder

**Figure 2:**
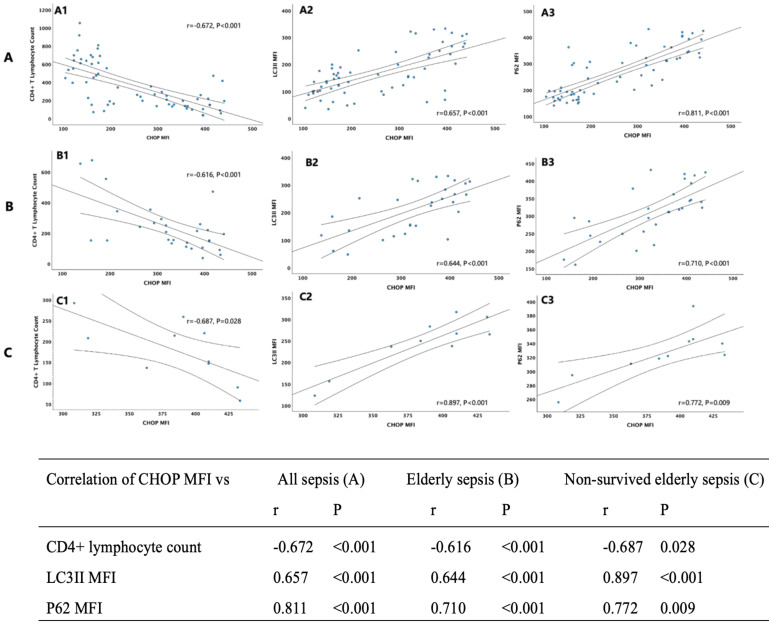
Nine scatter plots in three rows (A, B, C) with three columns each (1, 2, 3), showing correlations between CHOP MFI and CD4+ lymphocyte count, LC3II MFI, and P62 MFI across three sepsis categories: all sepsis, elderly sepsis, and non-survived elderly sepsis. Correlation coefficients (r) and p-values indicate significant relationships, detailed in an accompanying table.

The percentage of CD4^+^ lymphocytes expressing CHOP, and CHOP MFI on CD4^+^ cells were significantly higher in elderly sepsis patients than non-elderly ones (95.5% versus 81.1%, P=0.004; and 334.4 versus 164.2, P<0.0001, respectively), and at the same time the MFIs of mTOR, P62, and LC3II on CD4^+^ T lymphocyte were also significantly higher. When it comes to the comparison between survivors and non-survivors of all the sepsis patients, the differences of ERS and mTOR mediated autophagic-lysosomal disorder markers were still significant. Thereafter, we hypothesized that mTOR pathway mediated autophagic-lysosomal disorder might be significantly associated with differing expression levels of CHOP on CD4^+^ lymphocytes, and the overexpression of CHOP might be statistically correlated with CD4+ T lymphocyte reduction. Consistent with this hypothesis, Pearson’s analysis were performed and demonstrated that CHOP MFI on CD4^+^ lymphocytes was negatively correlated with CD4+ T lymphocyte count and positively significantly correlated with LC3II and P62 expression in all the sepsis patients (r=-0.672, P<0.001; r=0.657, P<0.0001 and r=0.811, P<0.001 respectively), elderly sepsis patients (r=-0.616, P<0.001; r=0.644, P<0.0001 and r=0.710, P<0.001 respectively), and non-survived elderly sepsis patients (r=-0.687, P=0.028; r=0.897, P<0.001 and r=0.772, P=0.00 respectively) (**Figure 2**).

## Discussion

This clinical study demonstrates that CHOP and GRP78 expression on CD4+ T lymphocyte is significantly higher in sepsis patients, indicating that ERS was much more enhanced. This phenomenon is more pronounced in elderly sepsis and non-survived ones. Meanwhile, CHOP MFI showed statistically correlation with MFIs of LC3II and P62 not only in sepsis patients, but also in elderly and non-survived sepsis patients, suggesting a strong association between ERS and autophagic-lysosomal disorder.

Sepsis is a major public health concern, particularly in the elderly, who are more susceptible to sepsis and exhibit high associated mortality rate. Timely recognition of sepsis in elderly individuals is challenging due to atypical presentations and delayed diagnosis (6). Meanwhile, sepsis could easily progress into SAI in elderly patients which further worsen the outcome (26). In this clinical study we found that elderly sepsis patients had significantly lower lymphocyte count, CD4+ T lymphocyte count and CD8+ T lymphocyte counts, along with enhanced ERS on CD4+ T cell. This suggests that ERS may contribute to SAI and sepsis progression.

ERS is a well-documented phenomenon in sepsis. When sepsis occurred, there is a mass of unfolded or misfolded proteins accumulated in cells. ER is a vital organelle responsible for protein translation, processing and modification. The waste accumulation in sepsis state disrupts ER homeostasis and causes ERS (11). Sustained or prolonged ERS induce apoptosis (27). Besides, in elderly sepsis individuals ERS could be much fiercer because accumulation of waste products results from a variety of age-associated alterations and simultaneously waste removal is compromised through a variety of mechanisms (28). Our findings confirm that ERS is enhanced in elderly sepsis patients, and is linked to decreased lymphocyte count and poor prognosis. This phenomenon was also seen and much more obvious in non-survived elderly sepsis patients. therefore, interventions on enhanced ERS could provide potential immunotherapy target in elderly sepsis patients.

Sepsis in elderly patients remains a formidable clinical challenge, characterized by suboptimal responses to standard treatments and a high propensity for rapid progression to SAI and mortality. Emerging evidence suggests that ERS plays a pivotal role in this pathological process. Under sustained cellular stress, the accumulation of misfolded or unfolded proteins triggers ERS, which, when unresolved, culminates in apoptosis of immune cells, particularly CD4+ T lymphocytes (20, 29). Our findings demonstrate that elderly sepsis patients exhibit exacerbated ERS in CD4+ T cells, accompanied by decreased CD4+ T cell count, which likely contributes to their impaired immune response and poor clinical outcomes. Preventing ERS may relieve the inflammatory state and damage to tissues and organs (30). However, no direct clinical interventions currently exist to mitigate its effects. Intriguingly, our pre-clinical work identified the mTOR-Akt signaling pathway as a critical regulator of ERS in sepsis (31). Furthermore, autophagic-lysosomal disorder modulated by mTOR pathway was proved to play vital roles in sepsis and SAI (26, 32). Therefore, interventions targeting ERS or the mTOR pathway could provide potential therapeutic strategies for elderly sepsis patients. The current study reveals significant correlations between the ERS marker-CHOP MFI and autophagic-lysosomal disorder markers-LC3II and P62 MFIs in CD4+ lymphocytes in sepsis patients, particularly in elderly sepsis and non-survived elderly ones. These findings underscore the mechanistic link between ERS and mTOR-mediated autophagic-lysosomal dysfunction, suggesting that therapeutic modulation of mTOR pathway or autophagic-lysosomal fusion could ameliorate enhanced ERS and improve outcomes.

Autophagy is a recycling process that is activated upon cellular stress and stimulates the degradation of the cellular components that are responsible for the derangement in cellular homeostasis. Therefore, it can be expected that autophagy may play a role in alleviating ER stress. Several studies indeed suggested that there is an important cross-talk between ER stress, the UPR and autophagy (12, 33–38). In human, age-related decline in autophagy-related gene expression and compromised autophagic flux contribute to the accumulation of protein aggregates and dysfunctional organelles, impaired pathogen clearance, and exacerbated inflammation-all of which may intensify ERS in elderly sepsis patients (39). The mTOR complex 1 played an important role in regulating autophagy, primarily functioning as an autophagy inhibitor (40). Therefore, pharmacological inhibition of mTOR signaling could rescue CD4+ T cell from ERS-induced apoptosis by restoring autophagosome-lysosome fusion. Rapamycin, an allosteric mTOR inhibitor, exerts its effect by interfering with substrate recruitment in the mTOR pathway. Notably, mTOR-targeted immunotherapy has demonstrated remarkable success in managing autoimmune disorders and malignancies, and has been gradually transitioned to clinical practice (41, 42). Therefore, using rapamycin to modulate the mTOR signaling pathway could regulate the onset of ERS-induced cell apoptosis through autophagy and ultimately reverse the fate of CD4+ T cells in elderly sepsis. Our study provided a potential intervention target for immunotherapy for elderly sepsis patients, which held significant clinical translational value.

There are several limitations in this study. First, this clinical research illustrated the significant association between ERS and mTOR pathway mediated autophagic-lysosomal disorder, but the detailed mechanisms are still unclear and need further study. In the future, animal experiments and clinical intervention studies will be designed to further validate the mechanism. Besides, the sample was related small, although the enhanced ERS has been able to show in elderly sepsis patients, and the sample size needs to be expanded to further confirm the conclusion of this study.

## Conclusions

ERS in CD4+ T cells is enhanced in sepsis patients, particularly in elderly and non-survived individuals. ERS was strongly associated with mTOR pathway mediated autophagic-lysosomal disorder.

## Data Availability

The original contributions presented in the study are included in the article/**Supplementary Material**. Further inquiries can be directed to the corresponding authors.
