# Supplementary material for: The endoplasmic reticulum stress status of CD4+ T lymphocytes and its association with mTOR-mediated autophagic-lysosomal disorder in elderly sepsis patients
Source: Front Immunol. 2025 Aug 26;16:1648075. doi: 10.3389/fimmu.2025.1648075 (PMC12417166; doi:10.3389/fimmu.2025.1648075)
Supplement: Supplementary file 2 [file DataSheet2.docx]

**The Endoplasmic Reticulum Stress Status of CD4+ T lymphocytes and Its Association with mTOR-mediated Autophagic-lysosomal Disorder in Elderly Sepsis Patients**

**Supplemental File S1** Enrollment flowchart

**Supplemental File S2** Comparison between sepsis and non-septic critically ill patients

**Supplemental File S3** Comparison of clinical parameters and treatment at ICU admission between elderly and non-elderly sepsis patients

**Supplemental file S4** Comparison of marker MFIs on CD4+ T lymphocytes between elderly and non-elderly sepsis patients

The MFI of CHOP, GRP78, LC3II, and P62 on CD4+ T lymphocytes were all significantly higher in elderly sepsis patients than in non-elderly sepsis patients (199.1 versus 139.2, P=0.026; 173.4 versus 103.8, P=0.023; 236.7 versus 123.5, P<0.001; 318.4 versus 192.5, P<0.001; 334.4 versus 164.2, P<0.001; 374.3 versus 218.6, P<0.001 respectively)

**Supplemental file S5** Comparison between survivors and non-survivors of all the sepsis patients based on in-hospital mortality

**Supplemental File S6** Comparison between survivors and non-survivors of elderly sepsis patients based on in-hospital mortality

**Supplemental File S1 Enrollment flowchart**

Critically ill patients admitted from December 1^st^ 2022 to June 30^th^ 2023 (N=1975)

Non-elderly Sepsis Patients (Age<65 years, N=33)

Elderly Sepsis Patients (Age≥65 years, N=29)

Excluding:

1. Age<18 years (N=15)
2. Died within 48 hours after ICU admission (N=3)
3. During treatment of malignant tumor (N=27)
4. Autoimmune Diseases receiving glucocorticoids and/or immunosuppressors (N=32)
5. Acquired immunodeficiency (N=4)
6. Failed to obtain consent form (N=5)
7. Lost follow-up (N=4)

Control group (N=17)

Non-septic Critically Ill Patients enrolled from June 1^st^ 2023 to June 30^th^ 2023 (N=17)

Meeting enrollment criteria of Infection and Sepsis

Sepsis patients screened out (N=152)

Enrolled Sepsis Patients (N=62)

**Supplemental File S2 Comparison between sepsis and non-septic critically ill patients**

|  | All (N=79) | Sepsis (N=62) | Non-sepsis (N=17) | P |
| --- | --- | --- | --- | --- |
| **Baseline characteristics** |  |  |  |  |
| Age | 63 (51, 70) | 63 (51, 71) | 63 (52, 66) | 0.340 |
| Sex (Male n, %) | 45 (57.0%) | 35 (56.5%) | 10 (58.8%) | 0.861 |
| Comorbidities  CHD  COPD  DM  CKD  CLD  Solid Tumor  Autoimmune Disease  Hematologic Disease | 37 (46.8%)  5 (6.3%)  24 (30.4%)  7 (8.9%)  5 (6.3%)  21 (26.6%)  10 (12.7%)  6 (7.6%) | 22 (35.5%)  5 (8.1%)  20 (32.3%)  7 (11.3%)  4 (6.5%)  17 (27.4%)  10 (16.1%)  3 (4.8%) | 15 (88.2%)  0  4 (23.5%)  0  1 (5.9%)  4 (23.5%)  0  1 (5.9%) | <0.001  0.226  0.488  0.147  0.932  0.748  0.076  0.764 |
| Apache II score | 18 (12, 26) | 19 (16, 28) | 8 (6, 12) | <0.001 |
| SOFA score | 7 (4, 11) | 8 (5, 11) | 5 (4, 6) | 0.011 |
| Hospital Stay | 12 (8, 19) | 10 (8, 16) | 19 (13.5, 23) | 0.001 |
| ICU Stay | 4 (3, 9) | 5 (3, 11) | 4 (2, 6) | 0.316 |
| In-hospital Mortality (n, %) | 13 (16.5%) | 13 (21%) | 0 | 0.039 |
| **Vital signs and biochemical tests at admission** | | | | |
| Temperature | 37.0 (36.4, 37.5) | 36.9 (36.3, 37.2) | 37.8 (36.9, 38.3) | <0.001 |
| Heart Rate | 89 (77, 98) | 87 (75, 98) | 91 (89, 96) | 0.257 |
| RR | 16 (15, 20) | 16 (14, 20) | 16 (15, 20) | 0.919 |
| P/F ratio | 340.0 (285.0, 424.0) | 332.0 (275.5, 428.0) | 366.7 (305.8, 420.3) | 0.672 |
| PaCO2 | 39.1 (35.2, 41.8) | 39.1 (35.2, 42.1) | 39.2 (35.5, 39.8) | 0.459 |
| Lactate | 3.3 (2.5, 4.4) | 3.6 (2.8, 4.9) | 2.0 (1.5, 2.7) | <0.001 |
| Platelet Count | 165 (104, 212) | 168 (93, 232) | 156 (131, 175) | 0.621 |
| Creatinine | 85.0 (57.0, 120.0) | 84.5 (52.5, 148.5) | 86.0 (68.5, 89.5) | 0.711 |
| Bilirubin | 17.6 (12.6, 29.8) | 16.9 (11.4, 29.9) | 20.9 (14.1, 27.6) | 0.482 |
| Albumin | 33 (31, 36) | 32 (30, 35) | 36 (34, 38) | <0.001 |
| Prothrombin Time | 13.6 (12.6, 16.1) | 13.8 (12.5, 15.3) | 13.5 (12.8, 14.2) | 0.703 |
| APTT-R | 1.1 (0.9, 1.3) | 1.2 (1.0, 1.4) | 1.0 (0.9, 1.1) | <0.001 |
| **T lymphocyte subsets (/ul)** |  |  |  |  |
| WBC | 11835 (7888, 15530) | 11835 (7785, 16820) | 11700 (8013, 14375) | 0.520 |
| Monocyte Count | 550 (320, 740) | 450 (250, 710) | 650 (515, 790) | 0.068 |
| Lymphocyte Count | 637 (430, 1158) | 625 (392, 1171) | 738 (530, 1161) | 0.346 |
| B Lymphocyte | 119 (63, 210) | 116 (63, 219) | 120 (76, 192) | 0.900 |
| NK T cell Count | 65 (37, 108) | 57 (32, 93) | 93 (70, 151) | 0.002 |
| T Lymphocyte Count | 433 (278, 837) | 425 (261, 873) | 526 (334, 790) | 0.629 |
| CD4+ T cell Count | 261 (153, 537) | 260 (152, 567) | 287 (188, 423) | 0.811 |
| CD4+CD25+Treg (%) | 13.9 (10.5, 16.6) | 14.3 (11.9, 17.1) | 13.5 (6.8, 15.9) | 0.183 |
| CD4+CD25+ Treg Count | 37 (24, 69) | 38 (23, 75) | 34 (25, 62) | 0.616 |
| Fox3+Treg Count | 17 (10, 30) | 17 (10, 30) | 17 (12, 27) | 0.948 |
| Th1 Count | 15 (7, 33) | 14 (7, 34) | 18 (7, 39) | 0.322 |
| Th2 Count | 37 (22, 72) | 37 (22, 72) | 36 (25, 70) | 0.703 |
| Th17 Count | 22 (9, 38) | 24 (10, 38) | 13 (9, 57) | 0.765 |
| CD8+ T cell Count | 152 (105, 246) | 145 (89, 248) | 167 (134, 235) | 0.407 |
| CD4+CD28+ T cell | 249 (146, 514) | 242 (137, 553) | 279 (179, 442) | 0.788 |
| CD8+CD28+ T cell | 75 (36, 151) | 72 (32, 159) | 87 (64, 130) | 0.523 |
| CD8+CD38+ T cell | 71 (49, 127) | 64 (45, 131) | 77 (61, 120) | 0.637 |
| CD4+/CD8+ cell ratio | 1.8 (1.2, 2.9) | 1.8 (1.2, 2.9) | 1.8 (1.2, 2.4) | 0.659 |
| **Immunofluorescent staining results on CD4^+^ T cells** | | | | |
| mTOR (%) | 81.2 (60.5, 91.3) | 81.4 (60.2, 90.9) | 75.8 (59.6, 93.9) | 0.853 |
| mTOR MFI | 149.3 (115.9, 267.8) | 166.2 (114.4, 306.4) | 143.6 (127.5, 153.8) | 0.118 |
| LC3I (%) | 31.4 (13.4, 56.9) | 29.0 (12.9, 56.7) | 41.7 (18.6, 61.0) | 0.294 |
| LC3I MFI | 151.5 (90.3, 217.8) | 148.8 (84.6, 256.3) | 151.9 (125.7, 169.1) | 0.943 |
| LC3II (%) | 41.0 (22.5, 82.1) | 47.2 (25.6, 85.7) | 23.7 (6.3, 40.6) | 0.004 |
| LC3II MFI | 131.7 (99.1, 198.9) | 152.7 (100.9, 236.9) | 99.6 (64.5, 118.1) | <0.001 |
| P62 (%) | 95.7 (88.7, 97.6) | 95.7 (90.1, 97.6) | 94.5 (83.4, 97.6) | 0.642 |
| P62 MFI | 200.6 (173.7, 318.4) | 246.6 (183.7, 340.8) | 177.2 (139.6, 190.7) | <0.001 |
| CHOP (%) | 81.3 (66.7, 97.1) | 92.4 (74.3, 97.6) | 64.1 (58.5, 68.1) | <0.001 |
| CHOP MFI | 173.9 (142.9, 330.6) | 210.9 (159.7, 361.7) | 142.9 (133.2, 151.9) | <0.001 |
| GRP78 (%) | 85.3 (77.8, 95.3) | 88.0 (77.7, 96.1) | 80.5 (78.0, 91.3) | 0.168 |
| GRP78 MFI | 238.1 (199.3, 374.3) | 279.1 (198.7, 383.4) | 223.7 (197.8, 241.5) | 0.045 |

Values are presented as median and interquartile range (IQR) for continuous variables or as number of cases and percentage for categorical data.

CHD chronic heart disease, COPD chronic obstructive pulmonary disease, DM diabetes mellitus, CKD chronic kidney disease, CLD chronic liver disease, Apache II score, acute physiology and chronic health evaluation II score, SOFA score, subsequent organ failure assessment score, ICU, intensive care unit, RR respiratory rate, P/F ratio the ratio between partial fraction of arterial oxygen and fraction of inspiratory oxygen, PaCO2, partial fraction of arterial carbon deoxidate, APTT-R, activated partial thromboplastin time ratio, WBC white blood count, NK T cells, natural killer T cells, mTOR, mammalian target of rapamycin, LC3II, microtubule-associated protein light chain 3 type II, CHOP PERK-mediated C/EBP homologous protein, MFI, mean fluorescence intensity.

**Supplemental File S3 Comparison of clinical parameters and treatment at ICU admission between elderly and non-elderly sepsis patients**

|  | All Sepsis (N=62) | Elderly sepsis (N=29) | Non-elderly sepsis (N=33) | P | |
| --- | --- | --- | --- | --- | --- |
| **Clinical Parameters at ICU Admission** | | | | |  |
| Temperature | 36.9 (36.3, 37.2) | 36.8 (36.2, 37.0) | 37.0 (36.3, 37.5) | 0.055 | |
| Heart Rate | 87 (75, 98) | 82 (72, 88) | 96 (79, 101) | 0.002 | |
| Respiratory Rate | 16 (14, 20) | 16 (14, 20) | 17 (15, 21) | 0.130 | |
| P/F ratio | 332.0 (275.5, 428.0) | 319.0 (255.0, 424.0) | 352.0 (310.0, 435.0) | 0.313 | |
| PaCO2 | 39.1 (35.2, 42.1) | 38.0 (33.7, 41.6) | 40.5 (35.6, 42.3) | 0.204 | |
| Lactate | 3.6 (2.8, 4.9) | 3.7 (2.6, 5.2) | 3.6 (2.9, 4.4) | 0.977 | |
| ScvO2 | 74.8 (687.3, 81.3) | 73.2 (67.3, 80.4) | 75.4 (66.5, 81.7) | 0.657 | |
| Pv-aCO2 | 2.5 (1.4, 5.1) | 2.4 (1.3, 5.0) | 2.8 (2.0, 5.1) | 0.481 | |
| Platelet Count | 168 (93, 232) | 155 (89, 206) | 181 (91, 275) | 0.207 | |
| Creatinine | 84.5 (52.5, 148.5) | 100.0 (60.0, 180.5) | 83.0 (44.0, 128.0) | 0.352 | |
| Bilirubin | 16.9 (11.4, 29.9) | 17.1 (12.2, 27.8) | 16.7 (10.2, 33.8) | 0.821 | |
| Albumin | 32 (30, 35) | 32 (30, 35) | 32 (31, 35) | 0.650 | |
| Prothrombin Time | 13.8 (12.5, 15.3) | 14.0 (12.6, 16.4) | 13.3 (12.5, 14.6) | 0.062 | |
| APTT-R | 1.2 (1.0, 1.4) | 1.3 (1.0, 1.5) | 1.1 (1.0, 1.3) | 0.277 | |
| Procalcitonin | 3.9 (0.9, 19.3) | 7.3 (0.9, 24.5) | 2.7 (0.9, 16.5) | 0.204 | |
| G test | 26.8 (14.3, 69.6) | 28.9 (16.9, 78.7) | 18.8 (10.0, 68.1) | 0.109 | |
| GM | 0.1 (0.1, 0.2) | 0.1 (0.1, 0.2) | 0.1 (0.0, 0.2) | 0.539 | |
| hsCRP | 157.2 (80.9, 248.6) | 212.4 (86.2, 302.9) | 130.9 (72.3, 219.9) | 0.071 | |
| **Treatment at ICU Admission** | | | | |  |
| Mechanical Ventilation  Vasopressors  Hemodynamic Monitor  CRRT  ECMO | 55 (88.7%)  50 (80.6%)  7 (11.3%)  17 (27.4%)  2 (3.2%) | 26 (89.7%)  25 (86.2%)  3 (10.3%)  9 (31.0%)  1 (3.4%) | 29 (87.9%)  28 (84.8%)  4 (12.1%)  8 (24.2%)  1 (3.0%) | 0.825  0.880  0.825  0.550  0.926 | |
| **Initial antibiotics** |  |  |  |  | |
| Anti-Gram Positive | 28 (45.2%) | 13 (44.8%) | 15 (45.5%) | 0.961 | |
| Anti-Gram Negative | 55 (88.7%) | 26 (89.7%) | 29 (87.9%) | 0.825 | |
| Anti-fungal drugs | 12 (19.4%) | 6 (20.7%) | 6 (18.2%) | 0.803 | |
| Anti-Virus drugs | 5 (8.1%) | 2 (6.9%) | 3 (9.1%) | 0.752 | |

All the enrolled sepsis patients were divided into elderly (aged≥65 years) and non-elderly (aged<65 years) groups. Values are presented as median and interquartile range (IQR) for continuous variables or as number of cases and percentage for categorical data.

P/F ratio the ratio between partial fraction of arterial oxygen and fraction of inspiratory oxygen, PaCO2, partial fraction of arterial carbon deoxidate, APTT-R, activated partial thromboplastin time ratio, ScvO2 central venous oxygen saturation, Pv-aCO2 Venous-to-Arterial Carbon Dioxide Difference, hsCRP, hypersensitive C-reactive protein, CRRT, continuous renal replacement therapy, ECMO, extracorporeal membrane oxygenation.

**Supplemental file S4 Comparison of marker MFIs on CD4+ T lymphocytes between elderly and non-elderly sepsis patients**

**
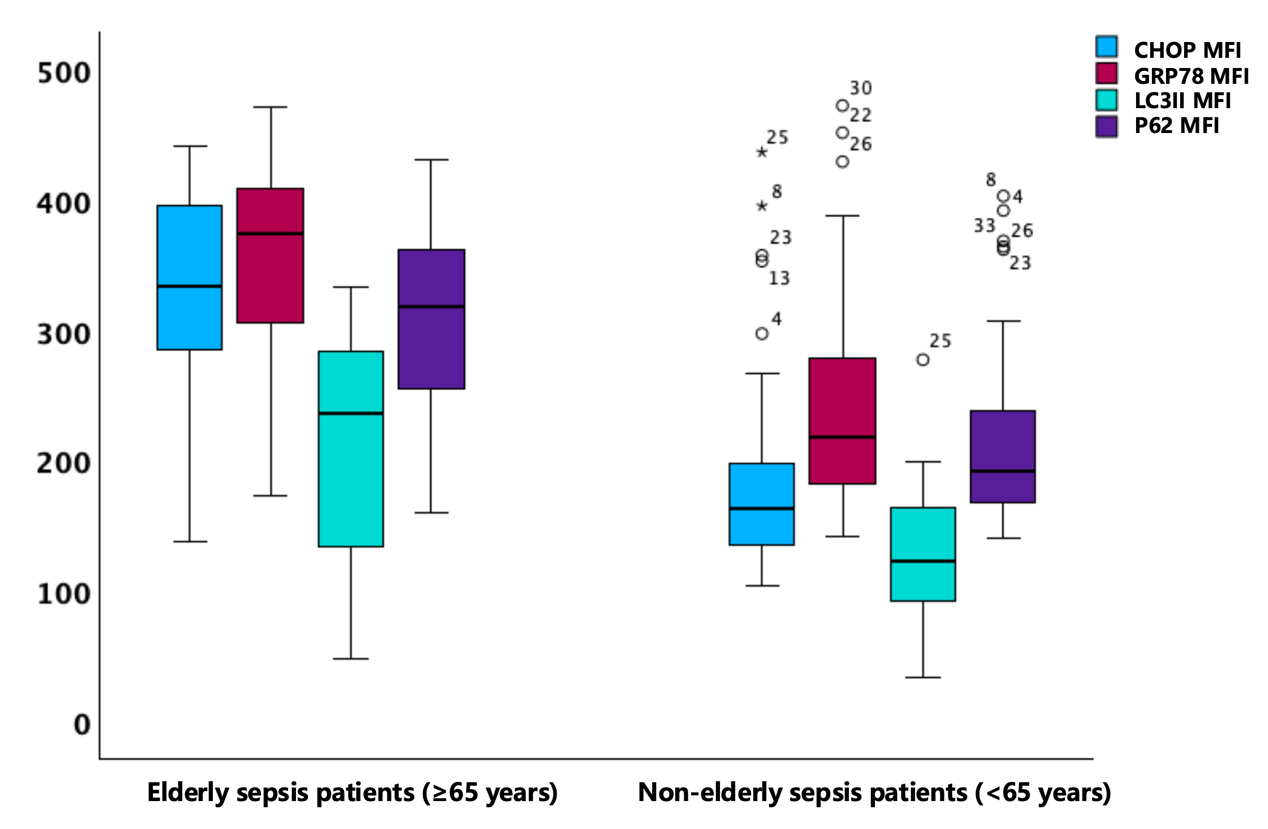
**

the MFI of CHOP, GRP78, LC3II, and P62 on CD4+ T lymphocytes were all significantly higher in elderly sepsis patients than in non-elderly sepsis patients (199.1 versus 139.2, P=0.026; 173.4 versus 103.8, P=0.023; 236.7 versus 123.5, P<0.001; 318.4 versus 192.5, P<0.001; 334.4 versus 164.2, P<0.001; 374.3 versus 218.6, P<0.001 respectively)

MFI mean fluorescent intensity, LC3II microtubule-associated protein light chain 3 type II, CHOP PERK-mediated C/EBP homologous protein.

**Supplemental file S5 Comparison between survivors and non-survivors of all the sepsis patients based on in-hospital mortality**

|  | All Sepsis (N=62) | Non-survivors (N=13) | Survivors* (N=49) | P |  |
| --- | --- | --- | --- | --- | --- |
| **Baseline characteristics** |  |  |  |  |  |
| Age | 63 (51, 71) | 70 (65, 74) | 60 (51, 70) | 0.027 |  |
| Age (>=65years) | 29 (46.8%) | 10 (76.9%) | 19 (38.8%) | 0.014 |  |
| Sex (Male n, %) | 35 (56.5%) | 8 (61.5%) | 27 (55.1%) | 0.677 |  |
| Transferred from  Ward  ER | 9 (14.5%)  53 (85.5%) | 2 (15.4%)  11 (84.6%) | 7 (14.3%)  42 (85.7%) | 0.920 |  |
| Comorbidities  CHD  COPD  DM  CKD  CLD  Solid Tumor  Autoimmune Disease  Hematologic Disease | 22 (35.5%)  5 (8.1%)  20 (32.3%)  7 (11.3%)  4 (6.5%)  17 (27.4%)  10 (16.1%)  3 (4.8%) | 9 (69.2%)  1 (7.7%)  6 (46.2%)  4 (30.8%)  1 (7.7%)  3 (23.1%)  1 (7.7%)  2 (15.4%) | 13 (26.5%)  4 (8.2%)  14 (28.6%)  3 (6.1%)  3 (6.1%)  14 (28.6%)  9 (18.4%)  3 (6.1%) | 0.004  0.956  0.228  0.013  0.838  0.693  0.352  0.276 |  |
| Infection Sites  Pulmonary  BSI  Abdominal  SSTI | 34 (54.8%)  2 (3.2%)  23 (37.1%)  3 (4.8%) | 7 (53.8%)  0  5 (38.5%)  1 (7.7%) | 27 (55.1%)  2 (4.1%)  18 (36.7%)  2 (4.1%) | 0.845 |  |
| Apache II score | 19 (16, 28) | 30 (26, 34) | 19 (16, 24) | <0.001 |  |
| SOFA score | 8 (5, 11) | 11 (9, 13) | 7 (4, 11) | <0.001 |  |
| CPIS score | 2 (0, 6) | 6 (2, 7) | 2 (0, 6) | 0.016 |  |
| Hospital Stay | 10 (8, 16) | 15 (11, 26) | 9 (7, 15) | 0.016 |  |
| ICU Stay | 5 (3, 11) | 8 (4, 12) | 4 (3, 9) | 0.156 |  |
| **Clinical Parameters at ICU admission** | | | | | |
| Temperature | 36.9 (36.3, 37.2) | 36.2 (36.0, 37.1) | 37.0 (36.5, 37.3) | 0.022 |  |
| Heart Rate | 87 (75, 98) | 86 (74, 99) | 87 (75, 98) | 0.822 |  |
| RR | 16 (14, 20) | 16 (14, 19) | 17 (15, 21) | 0.152 |  |
| P/F ratio | 332.0 (275.5, 428.0) | 350.0 (279.0, 403.5) | 330.0 (271.5, 449.5) | 0.684 |  |
| PaCO2 | 39.1 (35.2, 42.1) | 40.0 (36.6, 45.1) | 39.0 (35.1, 41.9) | 0.416 |  |
| Lactate | 3.6 (2.8, 4.9) | 5.5 (4.9, 6.9) | 3.2 (2.7, 3.9) | 0.028 |  |
| ScvO2 | 74.8 (687.3, 81.3) | 73.6 (65.3, 83.5) | 74.9 (67.3, 80.6) | 0.986 |  |
| Pv-aCO2 | 2.5 (1.4, 5.1) | 2.5 (1.4, 4.7) | 2.5 (1.4, 5.1) | 0.706 |  |
| Platelet Count | 168 (93, 232) | 186 (56, 226) | 168 (96, 233) | 0.775 |  |
| Creatinine | 84.5 (52.5, 148.5) | 148.0 (107.5, 237.5) | 74.0 (47.5, 111.5) | 0.007 |  |
| Bilirubin | 16.9 (11.4, 29.9) | 16.9 (6.7, 27.5) | 16.9 (12.0, 30.7) | 0.622 |  |
| Albumin | 32 (30, 35) | 32 (29, 34) | 32 (31, 36) | 0.309 |  |
| Prothrombin Time | 13.8 (12.5, 15.3) | 16.3 (12.6, 17.4) | 13.6 (12.5, 14.7) | 0.046 |  |
| APTT-R | 1.2 (1.0, 1.4) | 1.3 (1.0, 1.4) | 1.2 (1.0, 1.4) | 0.640 |  |
| Procalcitonin | 3.9 (0.9, 19.3) | 13.0 (2.3, 25.0) | 3.0 (0.8, 17.5) | 0.116 |  |
| G test | 26.8 (14.3, 69.6) | 50.5 (23.5, 90.5) | 20.0 (11.6, 61.2) | 0.079 |  |
| GM | 0.1 (0.1, 0.2) | 0.1 (0.1, 0.2) | 0.1 (0.1, 0.2) | 0.659 |  |
| hsCRP | 157.2 (80.9, 248.6) | 156.3 (72.9, 297.5) | 158.1 (80.4, 243.5) | 0.628 |  |
| C3 | 0.8 (0.7, 1.0) | 0.7 (0.6, 1.0) | 0.9 (0.7, 1.0) | 0.027 |  |
| C4 | 0.2 (0.1, 0.2) | 0.2 (0.1, 0.3) | 0.2 (0.1, 0.2) | 0.795 |  |
| IgG | 7.9 (5.6, 10.6) | 7.9 (6.4, 9.3) | 7.9 (4.9, 11.4) | 0.836 |  |
| IgA | 2.1 (1.3, 2.9) | 2.6 (1.5, 2.9) | 1.9 (1.3, 2.9) | 0.511 |  |
| IgM | 0.6 (0.4, 0.9) | 0.6 (0.4, 0.8) | 0.6 (0.4, 0.9) | 0.729 |  |
| IL-6 | 75.8 (34.5, 334.3) | 299.0 (68.8, 1000.0) | 52.6 (31.3, 133.0) | 0.015 |  |
| IL-8 | 49.0 (30.8,128.5) | 148.0 (55.5, 380.5) | 45.0 (27.0, 97.5) | 0.005 |  |
| IL-10 | 7.9 (5.0, 19.0) | 14.6 (7.9, 38.9) | 6.2 (5.0, 17.4) | 0.061 |  |
| TNF-a | 19.1 (12.3, 29.2) | 19.4 (14.5, 49.2) | 18.2 (11.9, 26.2) | 0.069 |  |
| Initial Treatment  Mechanical Ventilation  Vasopressors  Hemodynamic Monitor  CRRT  ECMO | 55 (88.7%)  50 (80.6%)  7 (11.3%)  17 (27.4%)  2 (3.2%) | 13 (100%)  13 (100%)  4 (30.8%)  6 (46.2%)  0 | 42 (85.7%)  40 (81.6%)  3 (6.1%)  11 (22.4%)  2 (4.1%) | 0.148  0.095  0.013  0.089  0.459 |  |
| **T lymphocyte subsets (median and IQR) (/uL)** | | | | |  |
| WBC | 11835 (7785, 16820) | 14900 (6905, 17890) | 11790 (8305, 16725) | 0.917 |  |
| Monocyte Count | 450 (250, 710) | 430 (195, 1140) | 450 (305, 695) | 0.775 |  |
| Lymphocyte Count | 625 (392, 1171) | 454 (345, 593) | 731 (397, 1312) | 0.013 |  |
| B Lymphocyte | 116 (63, 219) | 71 (41, 168) | 126 (66, 253) | 0.061 |  |
| NK T cell Count | 57 (32, 93) | 23 (11, 53) | 60 (45, 100) | 0.008 |  |
| T Lymphocyte Count | 425 (261, 873) | 283 (199, 416) | 597 (286, 925) | 0.009 |  |
| CD4+ T cell Count | 260 (152, 567) | 152 (114, 240) | 394 (161, 626) | 0.005 |  |
| CD8+ T cell Count | 145 (89, 248) | 111 (55, 146) | 175 (95, 306) | 0.085 |  |
| CD4+CD28+ T cell | 242 (137, 553) | 146 (84, 225) | 353 (152, 582) | 0.006 |  |
| CD8+CD28+ T cell | 72 (32, 159) | 29 (14, 90) | 81 (37, 170) | 0.007 |  |
| CD8+CD38+ T cell | 64 (45, 131) | 56 (33, 98) | 66 (44, 140) | 0.161 |  |
| CD4+/CD8+ cell ratio | 1.8 (1.2, 2.9) | 1.5 (1.0, 2.7) | 1.9 (1.3, 2.9) | 0.392 |  |
| **Markers on CD4^+^ T cells (median and IQR)** | | | | |  |
| mTOR (%) | 81.4 (60.2, 90.9) | 70.3 (39.4, 82.4) | 85.6 (60.6, 92.6) | 0.048 |  |
| mTOR MFI | 166.2 (114.4, 306.4) | 179.9 (123.7, 258.9) | 164.9 (114.4, 334.8) | 0.979 |  |
| LC3I (%) | 29.0 (12.9, 56.7) | 22.2 (2.6, 57.7) | 31.4 (14.6, 57.8) | 0.329 |  |
| LC3I MFI | 148.8 (84.6, 256.3) | 185.5 (75.2, 297.2) | 142.8 (87.2, 263.4) | 0.373 |  |
| LC3II (%) | 47.2 (25.6, 85.7) | 85.2 (75.2, 297.2) | 41.6 (22.9, 75.4) | 0.013 |  |
| LC3II MFI | 152.7 (100.9, 236.9) | 249.9 (181.5, 280.4) | 134.8 (96.9, 184.7) | <0.001 |  |
| P62 (%) | 95.7 (90.1, 97.6) | 96.6 (95.3, 97.6) | 94.8 (85.6, 97.6) | 0.076 |  |
| P62 MFI | 246.6 (183.7, 340.8) | 323.7 (306.5, 357.5) | 200.6 (174.2, 309.1) | 0.002 |  |
| CHOP (%) | 92.4 (74.3, 97.6) | 98.3 (95.6, 98.9) | 86.5 (71.5, 96.2) | <0.001 |  |
| CHOP MFI | 210.9 (159.7, 361.7) | 390.8 (313.6, 420.9) | 177.6 (148.9, 321.1) | <0.001 |  |
| GRP78 (%) | 88.0 (77.7, 96.1) | 96.4 (84.1, 98.0) | 85.3 (76.5, 95.1) | 0.035 |  |
| GRP78 MFI | 279.1 (198.7, 383.4) | 389.1 (311.2, 433.1) | 227.0 (191.6, 370.9) | <0.001 |  |

* All the sepsis patients were divided into survivors and non-survivors based on in-hospital mortality. Values are presented as median and interquartile range (IQR) for continuous variables or as number of cases and percentage for categorical data.

ER emergency room, CHD chronic heart disease, COPD chronic obstructive pulmonary disease, DM diabetes mellitus, CKD chronic kidney disease, CLD chronic liver disease, BSI bloodstream infection, SSTI skin and soft tissue infection, Apache II score, acute physiology and chronic health evaluation II score, SOFA score, subsequent organ failure assessment score, CPIS score clinical pulmonary infection score, ICU, intensive care unit, RR respiratory rate, P/F ratio the ratio between partial fraction of arterial oxygen and fraction of inspiratory oxygen, PaCO2, partial fraction of arterial carbon deoxidate, ScvO2 central venous oxygen saturation, Pv-aCO2 Venous-to-Arterial Carbon Dioxide Difference, APTT-R, activated partial thromboplastin time ratio, hsCRP, hypersensitive C-reactive protein, Ig immunoglobulin, IL interleukin, TNF-α, tumor necrosis factor α, CRRT, continuous renal replacement therapy; ECMO, extracorporeal membrane oxygenation, WBC white blood count, NK T cells, natural killer T cells, mTOR, mammalian target of rapamycin, LC3II, microtubule-associated protein light chain 3 type II, CHOP PERK-mediated C/EBP homologous protein, MFI, mean fluorescence intensity.

**Supplemental File S6 Comparison between survivors and non-survivors of elderly sepsis patients based on in-hospital mortality**

|  | All elderly sepsis (N=29) | Non-survivors (n=10) | Survivors* (n=19) | P | |
| --- | --- | --- | --- | --- | --- |
| **Baseline characteristics** |  |  |  |  | |
| Age (years) | 71 (69, 75) | 71 (70, 75) | 73 (69, 75) | 0.700 | |
| Sex (Male n, %) | 15 (51.7%) | 5 (50%) | 10 (52.6%) | 0.893 | |
| Transferred from  Ward  Emergency Room | 5 (17.2%)  24 (82.8%) | 2 (20%)  8 (80%) | 3 (15.8%)  16 (84.2%) | 0.775 | |
| Comorbidities  Chronic heart disease  COPD  Diabetes Mellitus  Chronic kidney disease  Chronic liver disease  Solid Tumor  Autoimmune Disease  Hematologic Disease | 10 (34.5%)  3 (10.3%)  12 (41.4%)  6 (20.7%)  2 (6.9%)  7 (24.1%)  7 (24.1%)  3 (10.3%) | 6 (60%)  1 (10%)  5 (50%)  4 (40%)  1 (10%)  2 (20%)  1 (10%)  2 (20%) | 4 (21.1%)  2 (10.5%)  7 (36.8%)  2 (10.5%)  1 (5.3%)  5 (26.3%)  6 (31.6%)  1 (5.3%) | 0.036  0.965  0.494  0.063  0.632  0.706  0.197  0.215 | |
| Infection Sites  Pulmonary  Bloodstream infection  Abdominal  SSTI | 16 (55.2%)  2 (6.9%)  9 (31.0%)  2 (6.9%) | 5 (50%)  0  4 (40%)  1 (10%) | 11 (57.9%)  2 (10.5%)  5 (26.3%)  1 (5.3%) | 0.629 | |
| Apache II score | 24 (17, 30) | 30 (26, 34) | 19 (16, 28) | 0.016 | |
| SOFA score | 10 (7, 12) | 11 (10, 13) | 8 (5, 11) | 0.064 | |
| Hospital Stay (days) | 14 (10, 24) | 15 (13, 27) | 12 (10, 21) | 0.270 | |
| ICU Stay (days) | 7 (4, 13) | 11 (4, 12) | 5 (4, 13) | 0.064 | |
| **Clinical Parameters at ICU Admission** | | | | |  |
| Temperature | 36.8 (36.2, 37.0) | 36.2 (36.0, 36.6) | 36.9 (36.5, 37.0) | 0.126 | |
| Heart Rate | 82 (72, 88) | 85 (72, 95) | 77 (71, 88) | 0.450 | |
| Respiratory Rate | 16 (14, 20) | 16 (13, 20) | 16 (14, 21) | 0.449 | |
| P/F ratio | 319.0 (255.0, 424.0) | 355.0 (282.0, 422.0) | 298.0 (243.0, 510.0) | 0.450 | |
| PaCO2 | 38.0 (33.7, 41.6) | 38.1 (34.5, 44.0) | 36.7 (33.3, 41.2) | 1.000 | |
| Lactate | 3.7 (2.6, 5.2) | 5.4 (4.7, 6.9) | 2.8 (2.5, 3.7) | <0.001 | |
| ScvO2 | 73.2 (67.3, 80.4) | 75.5 (67.6, 83.6) | 71.1 (67.3, 77.9) | 0.695 | |
| Pv-aCO2 | 2.4 (1.3, 5.0) | 2.0 (1.1, 3.7) | 2.8 (1.4, 5.2) | 0.695 | |
| Platelet Count | 155 (89, 206) | 134 (34, 205) | 155 (116, 217) | 1.000 | |
| Creatinine | 100.0 (60.0, 180.5) | 180.5 (110.7, 259.8) | 71.0 (47.0, 106.0) | 0.002 | |
| Bilirubin | 17.1 (12.2, 27.8) | 31.0 (28.0, 33.2) | 17.1 (11.2, 28.8) | 1.000 | |
| Albumin | 32 (30, 35) | 31 (28, 33) | 33 (30, 36) | 0.700 | |
| Prothrombin Time | 14.0 (12.6, 16.4) | 16.4 (13.2, 18.2) | 13.8 (12.5, 15.3) | 0.128 | |
| APTT-R | 1.3 (1.0, 1.5) | 1.3 (1.1, 1.4) | 1.3 (1.0, 1.5) | 0.450 | |
| Procalcitonin | 7.3 (0.9, 24.5) | 10.2 (2.6, 23.8) | 4.0 (0.6, 30.0) | 1.000 | |
| G test | 28.9 (16.9, 78.7) | 43.1 (20.9, 110.1) | 28.7 (16.6, 68.8) | 0.450 | |
| GM | 0.1 (0.1, 0.2) | 0.1 (0.1, 0.2) | 0.1 (0.1, 0.2) | 0.694 | |
| hsCRP | 212.4 (86.2, 302.9) | 178.2 (81.9, 314.1) | 213.6 (81.4, 299.2) | 0.700 | |
| **Treatment at ICU Admission** |  |  |  |  | |
| Mechanical Ventilation  Vasopressors  Hemodynamic Monitor  CRRT  ECMO | 26 (89.7%)  25 (86.2%)  3 (10.3%)  9 (31.0%)  1 (3.4%) | 10 (100%)  10 (100%)  2 (20%)  5 (50%)  0 | 16 (84.2%)  15 (78.9%)  1 (5.3%)  4 (21.1%)  1 (5.3%) | 0.184  0.118  0.215  0.109  0.460 | |
| Initial antibiotics |  |  |  |  | |
| Anti-Gram Positive | 13 (44.8%) | 5 (50%) | 8 (42.1%) | 0.684 | |
| Anti-Gram Negative | 26 (89.7%) | 9 (90%) | 17 (89.5%) | 0.965 | |
| Anti-fungal drugs | 6 (20.7%) | 2 (20%) | 4 (21.1%) | 0.947 | |
| Anti-Virus drugs | 2 (6.9%) | 1 (10%) | 1 (5.3%) | 0.632 | |
| **Inflammatory factors (median and IQR)** | | | | | |
| C3 (g/L) | 0.8 (0.7, 1.0) | 0.7 (0.5, 0.9) | 0.9 (0.7, 1.1) | 0.050 | |
| C4 (g/L) | 0.2 (0.2, 0.2) | 0.2 (0.1, 0.3) | 0.2 (0.2, 0.2) | 1.000 | |
| IgG (g/L) | 7.9 (6.3, 11.6) | 7.1 (6.1, 9.7) | 8.4 (6.4, 12.8) | 0.433 | |
| IgA (g/L) | 2.3 (1.5, 2.7) | 2.6 (1.5, 2.9) | 1.9 (1.4, 2.7) | 0.450 | |
| IgM (g/L) | 0.6 (0.4, 0.7) | 0.7 (0.5, 0.8) | 0.4 (0.4, 0.7) | 0.450 | |
| IL-6 (pg/ml) | 128.0 (45.9, 674.0) | 296.5 (83.1, 676.0) | 96.9 (30.6, 780.0) | 0.128 | |
| IL-8 (pg/ml) | 62.0 (34.0, 161.0) | 116.5 (58.8, 315.5) | 49.0 (27.0, 130.0) | 0.128 | |
| IL-10 (pg/ml) | 10.6 (5.0, 31.7) | 15.7 (7.9, 35.1) | 6.5 (5.0, 32.1) | 0.450 | |
| TNF-a (pg/ml) | 19.4 (13.8, 34.1) | 19.3 (15.0, 46.2) | 20.6 (13.1, 31.9) | 0.700 | |
| **T lymphocyte subsets (median and IQR) (/uL)** | | | | | |
| White Blood Cell Count | 7820 (7085, 15935) | 15085 (7047, 17355) | 9030 (6980, 11990) | 0.450 | |
| Monocyte Count | 380 (205, 595) | 345 (208, 1135) | 380 (190, 580) | 1.000 | |
| Lymphocyte Count | 448 (365, 647) | 433 (285, 592) | 476 (380, 719) | 0.700 | |
| B Lymphocyte | 79 (41, 123) | 63 (34, 157) | 86 (49, 119) | 0.245 | |
| NK T cell Count | 51 (17, 76) | 21 (5, 77) | 55 (30, 86) | 0.245 | |
| T Lymphocyte Count | 326 (224, 444) | 272 (174, 414) | 342 (229, 477) | 0.700 | |
| CD4+ T cell Count | 194 (135, 279) | 180 (125, 230) | 194 (133, 354) | 1.000 | |
| CD8+ T cell Count | 107 (77, 135) | 108 (31, 145) | 107 (80, 125) | 0.714 | |
| CD4+CD28+ T cell | 180 (105, 279) | 163 (85, 212) | 190 (108, 339) | 0.700 | |
| CD8+CD28+ T cell | 35 (19, 68) | 28 (12, 79) | 43 (24, 69) | 0.245 | |
| CD4+/CD8+ cell ratio | 1.8 (1.4, 3.3) | 1.6 (1.3, 3.2) | 2.1 (1.4, 3.3) | 0.700 | |
| **Markers on CD4^+^ T cells (median and IQR)** | | | | | |
| mTOR (%) | 81.3 (62.9, 90.3) | 67.4 (17.7, 77.9) | 87.4 (79.9, 93.5) | 0.005 | |
| mTOR MFI | 199.1 (140.9, 334.8) | 148.0 (108.4, 191.3) | 303.3 (167.5, 360.4) | 0.050 | |
| LC3I (%) | 22.2 (9.7, 40.7) | 11.5 (2.1, 33.9) | 26.9 (17.4, 53.6) | 0.245 | |
| LC3I MFI | 173.4 (124.9, 263.4) | 147.7 (72.2, 227.1) | 178.8 (151.5, 284.9) | 0.700 | |
| LC3II (%) | 71.1 (34.2, 91.0) | 87.0 (62.6, 90.9) | 66.5 (29.9, 91.9) | 0.128 | |
| LC3II MFI | 236.7 (128.8, 294.4) | 257.5 (216.6, 288.8) | 185.4 (114.1, 312.5) | 0.128 | |
| P62 (%) | 96.2 (93.7, 97.6) | 96.5 (95.5, 97.3) | 95.7 (90.3, 98.3) | 0.450 | |
| P62 MFI | 318.4 (252.5, 369.9) | 322.9 (306.8, 343.9) | 0 (226.4, 406.4) | 0.450 | |
| CHOP (%) | 95.5 (88.2, 98.2) | 98.2 (95.9, 98.8) | 93.2 (79.9, 97.4) | 0.021 | |
| CHOP MFI | 334.4 (274.3, 401.4) | 398.7 (352.0, 415.4) | 318 (194.2, 372.5) | 0.021 | |
| GRP78 (%) | 90.2 (82.9, 96.9) | 96.9 (88.1, 98.2) | 86.8 (77.8, 94.8) | 0.128 | |
| GRP78 MFI | 374.3 (305.9, 418.4) | 418.5 (363.8, 443.5) | 329.0 (230.5, 381.9) | 0.021 | |

* All the elderly sepsis patients were divided into survivors and non-survivors based on in-hospital mortality. Values are presented as median and interquartile range (IQR) for continuous variables or as number of cases and percentage for categorical data.

ER emergency room, CHD chronic heart disease, COPD chronic obstructive pulmonary disease, DM diabetes mellitus, CKD chronic kidney disease, CLD chronic liver disease, BSI bloodstream infection, SSTI skin and soft tissue infection, Apache II score, acute physiology and chronic health evaluation II score, SOFA score, subsequent organ failure assessment score, CPIS score clinical pulmonary infection score, ICU, intensive care unit, RR respiratory rate, P/F ratio the ratio between partial fraction of arterial oxygen and fraction of inspiratory oxygen, PaCO2, partial fraction of arterial carbon deoxidate, ScvO2 central venous oxygen saturation, Pv-aCO2 Venous-to-Arterial Carbon Dioxide Difference, APTT-R, activated partial thromboplastin time ratio, hsCRP, hypersensitive C-reactive protein, Ig immunoglobulin, IL interleukin, TNF-α, tumor necrosis factor α, CRRT, continuous renal replacement therapy; ECMO, extracorporeal membrane oxygenation, WBC white blood count, NK T cells, natural killer T cells, mTOR, mammalian target of rapamycin, LC3II, microtubule-associated protein light chain 3 type II, CHOP PERK-mediated C/EBP homologous protein, MFI, mean fluorescence intensity.
